# Clinical outcomes of two patients with a novel pathogenic variant in *ASNS*: response to asparagine supplementation and review of the literature

**DOI:** 10.1038/s41439-019-0055-9

**Published:** 2019-05-22

**Authors:** Rosanne Sprute, Didem Ardicli, Kader Karli Oguz, Anna Malenica-Mandel, Hülya-Sevcan Daimagüler, Anne Koy, Turgay Coskun, Haicui Wang, Meral Topcu, Sebahattin Cirak

**Affiliations:** 1 Faculty of Medicine and University Hospital Cologne, Department of Pediatrics, University of Cologne, 50931 Cologne, Germany; 2 Faculty of Medicine and the Faculty of Mathematics and Natural Sciences, Center for Molecular Medicine Cologne (CMMC), University of Cologne, 50931 Cologne, Germany; 3 Department of Pediatric Neurology, Hacettepe University, 06100 Ankara, Turkey; 4 Department of Radiology, Hacettepe University, 06100 Ankara, Turkey; 5 Department of Pediatric Metabolism Unit, Hacettepe University, 06100 Ankara, Turkey

**Keywords:** Genetics research, Metabolic disorders, Paediatric neurological disorders

## Abstract

Asparagine synthetase deficiency (ASNSD, OMIM #615574) is a rare autosomal recessive neurometabolic inborn error that leads to severe cognitive impairment. It manifests with microcephaly, intractable seizures, and progressive cerebral atrophy. Currently, there is no established treatment for this condition. In our pediatric cohort, we discovered, by whole-exome sequencing in two siblings from Turkey, a novel homozygous missense mutation in asparagine synthetase at NM_133436.3 (*ASNS*_v001): c.1108C>T that results in an amino acid exchange p.(Leu370Phe), in the C-terminal domain. After identification of the metabolic defect, treatment with oral asparagine supplementation was attempted in both patients for 24 months. Asparagine supplementation was well tolerated, and no further disease progression was observed during treatment. One of our patients showed mild developmental progress with increased levels of attention and improved nonverbal communication. These results support our hypothesis that asparagine supplementation should be further investigated as a treatment option for ASNSD. We further reviewed all previously reported ASNSD cases with regard for their clinical phenotypes and brain imaging findings to provide an essential knowledge base for rapid diagnosis and future clinical studies.

## Amino acid deficiency: Asparagine supplementation for a neurometabolic disorder

Study of two cases with a rare metabolic disorder, asparagine synthetase deficiency (ASNSD), has identified a new mutation and a potential treatment. Healthy individuals produce the amino acid asparagine, but patients with ASNSD cannot, resulting in severe seizures and cognitive impairment. Sequencing all protein-coding genes, Sebahattin Cirak, University of Cologne, Germany, and co-workers identified a novel mutation in two brothers with ASNSD. Only 31 cases have been reported, and there is no curative treatment available. This disorder can currently only be diagnosed by genetic testing. The disease symptoms seem not to progress once the patients were given an oral asparagine supplementation. The researchers emphasize to be cautios since this is an anectodal experience, and note that newborns with seizures should be genetically tested and further controlled clinical efficacy trials are required.

## Introduction

Asparagine synthetase deficiency (ASNSD, OMIM #615574) is a rare neurometabolic disorder for which the number of reported cases has recently expanded. Currently, this disease can only be diagnosed by genetic testing. Three causative mutations in the gene, which encodes asparagine synthetase (ASNS), were originally reported by Ruzzo et al^1^. The authors discovered that recessive mutations in *ASNS* are responsible for a severe neurological phenotype characterized by congenital or progressive microcephaly and developmental delay.

Although ASNS mutations have repeatedly been associated with the phenotypic characteristics of these patients, the pathophysiology of ASNSD is not well understood. ASNS catalyzes the synthesis of asparagine and glutamate from aspartate and glutamine in an ATP-dependent amidotransferase reaction. The impact on the cellular homeostasis of these reactants is currently under discussion^2,3^. Congenital and progressive microcephaly and simplified gyration in children with ASNSD indicate that significant brain damage occurs during embryonic development, suggesting that ASNS activity is critical for brain development, either due to the accumulation of substrates or a deficiency in its products^3^.

Unfortunately, no established treatment is available for patients with ASNSD apart from symptomatic anticonvulsive medication. While asparagine can be synthesized in healthy individuals and therefore is defined as a nonessential amino acid, is presumably becomes essential in patients with ASNSD due to their deficiency in ASNS^1^. Hence, supplementation of the lacking amino acid is the first logical therapeutic approach. To date, only one documented trial has explored treatment with asparagine supplementation in a single patient with ASNSD^4^. However, treatment had to be stopped after 27 days because the patient developed irritability, sleep disturbances, and his seizure frequency and severity increased.

In the current study, we describe the neurological phenotype associated with a novel *ASNS* mutation in two siblings. The mutation was identified by whole-exome sequencing (WES). Furthermore, we review all cases reported in the literature with regard for their clinical phenotype and imaging findings in the brain^1,5–17^.

Moreover, we elaborate on the long-term clinical development of these two siblings, who were treated with oral asparagine supplementation for 2 years.

## Materials and methods

### Genetic workup

After obtaining parental informed consent, WES was used to uncover the genetic cause of the syndrome in this family^18^. The genomic DNA of index patient 1 was extracted using standard methods and enriched using a SureSelect Human All Exon V6 enrichment kit (Agilent, CA, USA) according to the manufacturer’s best-practice protocol. Exome sequencing was performed on an Illumina High Seq 4000 Sequencer (Illumina, CA, USA) with 2 × 75 base pair reads. The mean coverage was 78×, 10× coverage was attained for 96.2%, and 20× coverage was achieved for 90.9% of target sequences. For further variant analysis, please see the Supplementary information.

## Results

### Case presentations

**Index patient 1** was a 5-year- and 9-month-old boy and the first child of consanguineous Turkish parents. Both parents had normal neurological examinations. The child was born at 39 weeks of gestation by spontaneous delivery after an uncomplicated pregnancy. Head circumference, length, and weight at birth were 33 cm (10th to 15th percentile, WHO child growth standards^19^), 48 cm (15th to 25th percentile), and 3100 g (25th to 50th percentile), respectively. Apgar scores were 8 at minute 1 and 9 at minute 5. The boy developed generalized tonic seizures at the age of two months, and an electroencephalogram (EEG) showed multifocal epileptiform activity (Supplementary Fig. 1). Initial therapy with phenobarbital was started, but the seizures continued with a frequency of once a week. Levetiracetam was administered as a second antiepileptic drug and reduced the frequency of seizures to once every 3 months. Valproic acid and clobazam were subsequently started at the age of 4.5 years due to ongoing seizures. The global development of the child was retarded. He was able to sit without support at 12 months of age (window of achievement 3.7–8.9 months, WHO Motor Development Study^20^) and started walking independently at 34 months old (window of achievement 8–17.1 months). He later developed behavioral abnormalities with irritability and agitation and autistic features. There was no evidence of a sensorineural hearing deficit. On an ophthalmological examination, he did not show any relevant pathological findings. He has frequent respiratory infections (>10 times/year).

On examination at 3.5 years of age, his weight, length, and head circumference were 12 kg (<3rd percentile), 98 cm (15th to 50th percentile), and 42 cm (<3rd percentile), respectively. He had axial hypotonia with reduced head control and spasticity of the limbs with hyperreflexia and extensor plantar responses. On the last clinical assessment performed at the age of 5.5 years, his weight, length, and head circumference were 16.3 kg (5th to 15th percentile), 106 cm (5th to 15th percentile), and 42.5 cm (<3th percentile), respectively. He was able to walk independently with a broad-based ataxic gait and showed stereotypic movements of his upper extremities. He had fine motor and coordination difficulties, especially when requested to grasp for small objects. His cognition was severely impaired: he was able to recognize his parents and follow simple commands but could not speak with meaningful words at an age of 5.5 years.

**Fig. 1:**
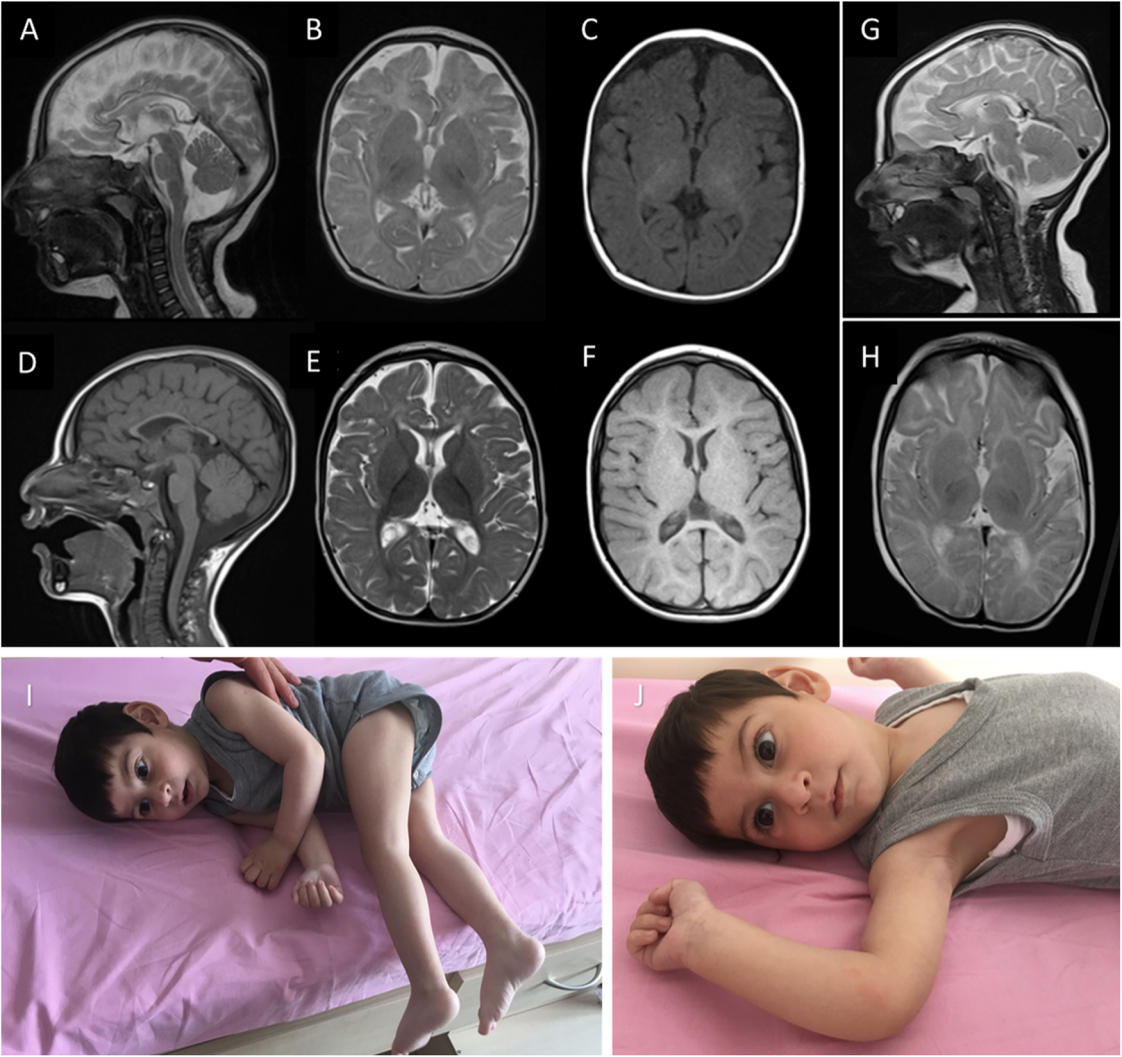
MRI findings and phenotypes of our patients with ASNSD. Brain MRI of patient 1 obtained at 4 months old (upper row, **a**–**c**) and 3.5 years old (lower row, **d**–**f**). Delayed myelination is best appreciated on the T1-weighted axial image obtained at 4 months old (**c**) and on the T2-weighted axial images obtained at 3.5 years old (**e**). The thin corpus callosum is displayed well in both sagittal T2- and T1-weighted images (**a**+**d**), while cerebral atrophy and the simplified gyration prominent in the frontal lobes were found on follow-up imaging (**d**–**f**). Brain MRI of patient 2 obtained at 4 months of age (**g**+**h**). The sagittal T2-weighted image (**g**) shows a very thin corpus callosum. Delayed myelination is better appreciated in image (**h**). The clinical phenotype of patient 2 (**i**+**j**) included absent fixation, reduced attention to external stimuli, and central hypotonia, which made him unable to sit without support

On neurometabolic investigation, an altered amino acid profile became evident (see section 3.3 Oral l-asparagine supplementation). Two brain magnetic resonance imagings (MRI) were performed when he was 4 months old and 3.5 years old and revealed microcephaly, delayed myelination, a thin corpus callosum, and cerebral atrophy of the frontal lobes (Fig. 1).

**Patient 2** was 3-year- and 9-month-old boy and the younger brother of patient 1. He was a full-term baby born by spontaneous uncomplicated delivery. His birth weight was 3000 g (25th percentile, WHO child growth standards^19^), and his head circumference was 31 cm (<3rd percentile). He manifested with a focal left-sided tonic seizure at 6 months of age. The seizures were well controlled by phenobarbital and levetiracetam, and he became seizure free in the first two years of life. Following a seizure recurrence at 2.5 years of age, phenobarbital treatment was replaced by clobazam. Currently, he has seizures once every 6 months. The EEG showed sharp and slow waves in the bilateral frontotemporal lobes (Supplementary Fig. 1).

The boy has experienced a similar developmental course with the same clinical phenotype as his brother. A brain MRI obtained at 4 months of age showed findings comparable to those found in his brother's imaging study, including severe microcephaly, delayed myelination, and a thin corpus callosum (Fig. 1). On an examination performed when he was 14 months old, his head circumference was 39 cm (<3rd percentile). On the latest assessment performed when he was 3.5 years old, his weight, height, and head circumference were 9.4 kg (<3rd percentile), 92 cm (<3rd percentile), and 39.5 cm (<3rd percentile), respectively. He was unable to sit independently and had limited eye contact, and reduced attention to external stimuli.

Currently, both siblings are on combined antiepileptic treatment, including combinations of levetiracetam (20 mg/kg/day), valproic acid (20 mg/kg/day), and clobazam (1 mg/kg/day) in patient 1 and levetiracetam (40 mg/kg/day) and clobazam (1 mg/kg/day) in patient 2. Patient 1 has also been treated with risperidone for behavioral problems since the age of 4.5 years.

### Whole-exome sequencing

**Fig. 2:**
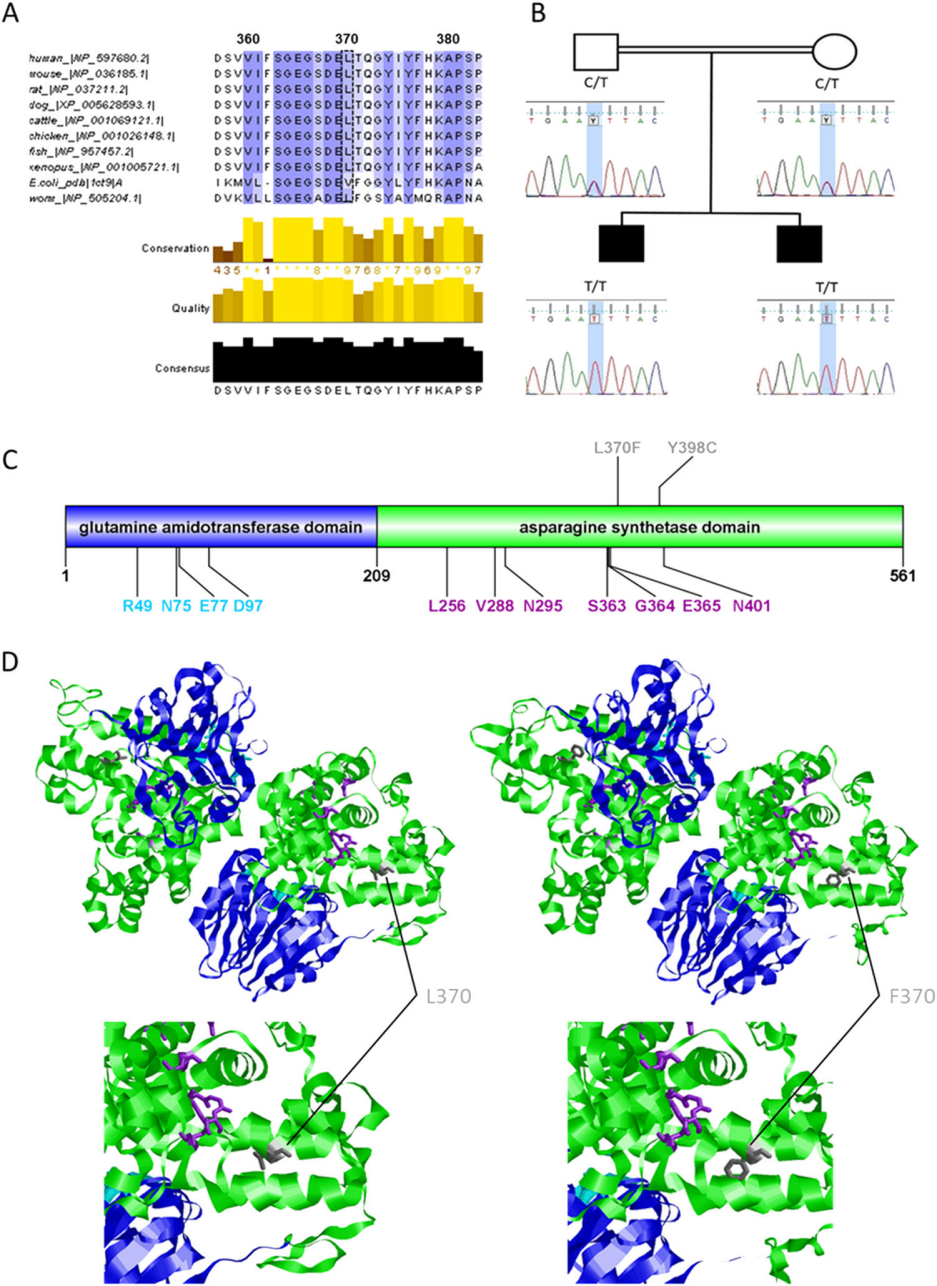
Conservation, cosegregation, and consequences of the mutated residue p.Leu370Phe. **a** Multiple alignment of human ASNS (NP_597680.2) with selected orthologues of mouse (NP_036185.1), rat (NP_037211.2), dog (XP_005628593.1), cattle (NP_001069121.1), chicken (NP_001026148.1), fish (NP_957457.2), *Xenopus* (NP_001005721.1), *Escherichia coli* (PDB 1CT9.1. A), and worm (NP_505204.1) shows that a highly conserved amino acid region is affected. Amino acid color labels were selected for the block substitution matrix 62. **b** Pedigree and chromatograms of the DNA sequence changes observed in the *ASNS* gene. Sanger sequencing revealed that both parents harbor a heterozygous variant at position c.1108C>T in *ASNS* (NM_133436.3). Both children are homozygous for this variant. **c** Linear model of the structure of the human ASNS protein. Blue: N-terminal domain containing the binding pocket for glutamine. Green: C-terminal domain containing the ATP-binding site. Cyan: Residues forming the glutamine-binding pocket. Purple: Residues for ATP binding through hydrogen bonds^2^. Gray: Localization of amino acid changes in reported patients who received oral asparagine supplementation^4,6^. **d** 3D structure model of ASNS, including wild type (UniProt ID P08243) and mutant p.Leu370Phe, by SWISS-MODEL using the crystal structure of asparagine synthetase B from *Escherichia coli* (PDB 1CT9.1.A)^25^. Gray: The affected amino acid p. Leu370 and the mutated amino acid p.Phe370 are located in the asparagine synthetase domain. Cyan: Residues p.Arg49, p.Asn75, p.Glu77, and p.Asp97 are important for glutamine binding. Purple: Amino acids p.Leu256, p.Val288, p.Asn295, p.Ser363, p.Gly364, and p.Glu365 are proposed as representing a binding site for ATP through hydrogen bonds^2^.

WES was performed to identify the pathogenic variant in the index patient 1. A missense mutation in ASNS was found in the homozygous and rare functional variants: NM_133436.3 (*ASNS*_v001): c.1108C>T; p.(Leu370Phe). This mutation is located in a highly conserved region of the C-terminal domain of the protein, which contains the ASNS domain (Fig. 2a, c, d)^2,14^. The variant was not found in the Genome Aggregation Database (http://gnomad.broadinstitute.org/), the Exome Aggregation Consortium (http://exac.broadinstitute.org/), or ClinVar (https://www.ncbi.nlm.nih.gov/clinvar/). The change of leucine to phenylalanine may have a significant effect on the enzyme’s activity. In silico prediction performed with online tools revealed that this mutation is ‘disease causing’ according to MutationTaster, the PROVEAN protein prediction tool predicted that the mutation is ‘deleterious’, and MutPred2 classified the mutation, which had a value of 0.84, as pathogenic (values > 0.5 suggest pathogenicity). We classified the variant as “likely pathogenic” according to the standards and guidelines of the American College of Medical Genetics and Genomics^21^.

Confirmation with the Sanger sequencing technique was implemented for both siblings and their parents. Patient 2 carried the same homozygous mutation in ASNS as his brother, and the consanguineous parents were both found to be heterozygous carriers of the variant (Fig. 2b).

### Oral l-asparagine supplementation

After identifying the diagnosis by WES, both patients were treated with oral l-asparagine supplementation, which was provided as powder extracted from 500 mg l-asparagine gelatin capsules, for 24 months, lasting from the age of 3 years and 9 months to 5 years and 9 months in patient 1 and from 21 months old to 3 years and 9 months old in patient 2. Asparagine supplementation was initially administered at a dosage of 50 mg/kg/day in 3 or 4 divided doses and was gradually increased up to 100 mg/kg/day. The parents gave their consent to this off-label trial due to the lack of established treatment options.

In patient 1, higher levels of attention, improvement in nonverbal communication, and increased interaction with the environment were reported by the parents during the course of the treatment. In patient 2, no clear improvement in psychomotor development was observed.

Neither the frequency nor the severity of seizures changed during asparagine supplementation. An EEG performed in patient 1 after 6 months of supplementation showed that there were fewer epileptiform discharges than were observed at baseline, but there was no further improvement after 21 months of treatment. A similar course was observed in the follow-up EEGs obtained in patient 2 (Supplementary Fig. 1).

Both patients' clinical courses stabilized during treatment, and we observed no further disease progression or developmental regression.

Before asparagine supplementation, the serum asparagine level in patient 1 was 3.0 µmol/l (reference range < 90 μmol/l, no lower-end value was available from the diagnostic laboratory). The serum aspartic acid values was elevated, at 34.02 µmol/l (reference range < 15 µmol/l). The serum glutamic acid level was 11 µmol/l, which was within the reference range (<75 µmol/l).

The level of asparagine increased slightly under supplementation (5.3, 4.4, and 3.5 µmol/l in the 6th, 12th, and 18th months of treatment, respectively).

The baseline cerebrospinal fluid (CSF) asparagine level was 2.91 µmol/l (no reference ranges available). Aspartic acid and glutamic acid levels were within the reference ranges. We sought to re-evaluate CSF amino acid levels by lumbar puncture to monitor changes due to treatment with asparagine, but this was denied by the parents.

Asparagine levels are naturally low in plasma, and CFS and asparagine levels in patients with ASNSD range between low and normal values; thus, their levels in plasma and CSF are not reliable as diagnostic markers^6,13,15,17^. Furthermore, reference values are inconsistent in the literature and determined by the laboratories. Scholl-Bürgi et al.^22^ measured the mean concentration of asparagine at 4.1 µmol/l with a standard deviation of 1.2 µmol/l in the CSF of 39 children older than 3 years of age. Akiyama et al.^23^ measured the mean as 3.1 µmol/l with a standard deviation of 2.6 µmol/l in 48 children older than 3 years old.

CSF asparagine levels were not detectable in 2/5 of the reported patients who underwent a CFS diagnostic^6,17^, but they were normal or only borderline-reduced in the other three cases^13,15,17^. Our index patient had a CSF asparagine concentration within the normal reference range according to the cited references^22,23^. In conclusion, ASNSD can be suspected on the basis of low values in plasma or CSF, but it cannot be ruled out by normal values. Thus, the diagnosis of ASNSD is still hampered by the lack of reliable biochemical test methods.

## Discussion

Recessive mutations in the *ASNS* gene have been shown to cause a neurometabolic syndrome with a neurodegenerative disease course^1,2^.

Since 2013, exome sequencing has led to the identification of 31 ASNSD cases in 21 unrelated families^1,5–17^. To date, 26 disease-causing variants have been identified in *ASNS*, with most of them due to recessive missense mutations in the C-terminal domain (Tables 1 and 2).

**Table 1:** Clinical comparison of our patients with 31 previously reported ASNSD cases^1,5–17^

									Neurological features							MRI findings	
Publication	Family	Ethnicity	ASNS mutation (new variant)	Patient number	Gender	Age at report	Birth weight [g]	Birth head circumference [cm]	Microcephaly	Abnormal EEG	Epilepsy (age of onset)	Hyperekplexia	Hyperreflexia	Axial hypotonia	Psychomotor delay	Decreased cerebral volume	Decreased size of pons and/or cerebellum
Ruzzo et al.^1^	1	Iranian Jews	c.1084T>G (1) p.F362V	1	M	14 y	NA	31.5	+	+	+(1 m)	−	+	−	+	+	+
	2	Iranian Jews	c.1084T>G p.F362V	2	M	14 y	NA	31	+	+	+(2 w)	−	+	−	+	+	+
				3	F	12 y	NA	31	+	+	+(3 w)	−	+	−	+	+	+
	3	Bangladeshi	c.1648 C>T (2) p.R550C	4	M	4 m	3400	31.5	+	+	−	+	+	−	+	+	+
				5	M	3 m	3520	33	+	+	−	+	+	+	+	+	+
				6	M	6 m	3230	32	+	+	−	+	+	+	+	+	+
	4	French Canadian	c.17C>A (3)/c.1648 C>T p.A6E/p.R550C	7	M	9 d	NA	31.5	NA	NA	+(4 d)	−	+	+	+	+	+
				8	M	11 m	NA	31	+	+	+(9 m)	−	+	+	+	+	+
				9	M	12 m	2160	28.5	+	+	+(8 d)	−	+	+	+	+	+
Alfadhel et al.^6^	5	Saudi Arabian	c.1193A>G (4) p.Y398C	10	M	5 y	3100	29.5	+	+	+(1 d)	−	+	+	+	+	+
				11	F	4 y	2650	26.5	+	+	+(1 d)	−	+	+	+	+	+
Ben Salem et al.^7^	6	Emirati	c.1193A>C (5) p.Y398C	12	M	5 y	3100	29.5	+	+	+(1 d)	−	+	NA	+	+	+
Palmer et al.^12^	7	Chinese/Brunei	c.866G>C (6)/c. 1010C>T (7) p.G289A/p. T337I	13	M	7 y	3340	32.5	+	+	+(1 m)	−	+	+	+	+	+
Reed et al.^8^	8	Yemeni	c.198_202delATATC (8) p.K66Nfs*10	14	F	3 m	2600	31	+	+	−	+	+	+	+	−	−
Sun et al.^24^	9	Indian	c.1019G>A (9) p.R340H	15	F	11 m	2220	30.5	+	+	−	−	+	+	+	+	+
				16	F	5 m	2240	28.5	NA	−	−	+	+	+	+	+	+
Seidahmed et al.^15^	10	Saudi Arabian	c.1219C>T (10) p. R407*	17	M	9 m	2675	29	+	+	+(NA)	+	+	−	NA	+	+
	11	Saudi Arabian	c.944A>G (11) p.Y315C	18	M	4 y	2790	29	+	+	−	+	+	−	+	+	+
Gataullina et al.^10^	12	NA	c.1439C>T (12)/c.1648 C>T p.S480F/p.R550C	19	M	8 m	3760	34	+	+	+(24 h)	−	NA	+	+	+	+
				20	F	8 m	NA	31	+	+	+(4 m)	+	NA	NA	+	+	+
Yamamoto et al.^17^	13	Japanese	c.434T>C (13)/c.740T>G (14) p.L145S/p.L247W	21	M	26 m	2408	29	+	+	+(7 m)	(+)	+	+	+	+	+
	14	Japanese	c.1466T>A (15)/c.1623–1624del (16) p.V489D/p.W541Cfs*	22	M	19 m	3122	33.4	+	+	+(3 m)	+	+	+	+	+	+
Gupta et al.^11^	15	Indian	c.1138G>T (17) p.A380S	23	F	2.5 y	NA	NA	+	+	+(3 m)	−	+	+	+	+	+
Abhyankar et al.^5^	16	NA	c.728T>C (18)/c.1097G>A (19) p.V243A/p. G366E	24	NA	15 m	NA	NA	+	NA	+(NA)	NA	NA	NA	+	+	+
Sacharow et al.^13^	17	Emirati	c.146G>A (20) p.R49Q	25	M	7 y	3400	34	+	+	+(6 m)	−	NA	−	+	+	NA
				26	F	4 y	3000	NA	+	+	+(6 m)	−	+	+	+	+	NA
Galada et al.^9^	18	Indian	c.1211G>A (21) p.R404H	27	M	10 d	2030	29.5	+	NA	+(24 h)	−	+	NA	NA	+	+
	19	Indian	c.224A>G (22)/c.413A>C (23) p.N75S/p.D138A	28	F	NA	2300	29	+	NA	−	−	+	NA	NA	+	+
	20	Indian	c.1649 G>A (24) p.R550H	29	M	13 m	2800	30	+	NA	+(24 h)	−	+	NA	+	+	+
Schleinitz et al.^14^	21	German	c.1165G>C (25)/c.601delA (26) p.E389Q/p.M201Wfs*28	30	F	19 y	3320	29.5	+	+	+(24 h)	−	+	+	+	+	+
				31	F	16 y	2450	30	+	+	+(1 m)	−	+	+	+	NA	NA
**This report**	**22**	**Turkish**	**c.1108C>T (27) p.L370F**	**32**	**M**	**5.5** **y**	**3100**	**33**	**+**	**+**	**+(2** **m)**	**−**	**+**	**+**	**+**	**+**	**−**
				**33**	**M**	**3.5** **y**	**3000**	**31**	**+**	**+**	**+(6** **m)**	**−**	**+**	**+**	**+**	**+**	**−**

Positions in the ASNS gene are annotated to NCBI RefSeq NM_133436.3. Amino acid positions are assigned using RefSeq NCBI NP_597680.2

*y* years, *m* months, *w* weeks, *d* days, *h* hours, *NA* not available

**Table 2:** Clinical phenotype of patients with ASNSD^1,5–17^

Gender (male: female)	65.6% male
	34.4% female
	(21:11)
Head circumference <10th percentile at birth	86.6% (26/30)
Congenital or progressive microcephaly	100% (31/31)
Mutation type (missense: nonsense: deletion: combined)	81.8% missense
	4.5% nonsense
	4.5% deletion
	9.1% combined
	(18:1:1:2)
Abnormal EEG	96.4% (27/28)
Epilepsy	75.8% (25/33)
Hyperekplexia	31.3% (10/32)
Hyperreflexia	100% (31/31)
Axial hypotonia	69.0% (20/29)
Psychomotor delay	100% (31/31)
Decreased cerebral volume on MRI	96.9% (31/32)
Decreased size of the pons and/or cerebellum in MRI	90.0% (27/30)

A list of variants, including those found in our siblings and all cases reported so far, was assembled and is shown in Table 1, which describes the major clinical symptoms and MRI findings observed in patients with ASNSD^1,5–17^.

Our cases show a similar clinical phenotype and findings in imaging as the majority of the so far reported patients (Table 2). Extracranial findings were present in only three of the reported cases (Table 1)^1,24^.

ASNS catalyzes the synthesis of asparagine and glutamate from aspartate and glutamine in an ATP-dependent amidotransferase reaction^2^. ASNS varies widely in its basal expression level but is present in almost all mammalian organs^2^. Structure-function studies performed on *Escherichia coli* AS-B (which uses glutamine as a nitrogen source) have revealed that two distinct catalytic domains are conserved in the human enzyme^25^. The N-terminal domain contains the glutamine-binding pocket for the amidotransferase reaction, while the C-terminal domain is the ASNS domain and contains the ATP-binding site (Fig. 2c, d)^2,14^.

In this study, we report a novel mutation in *ASNS* at position NM_133436.3 (*ASNS*_v001): c.1108C>T; p.(Leu370Phe) in two affected siblings in a consanguineous family. Models of the mutant ASNS protein (Fig. 2c, d) showed that the highly conserved amino acid position 370 is close to the catalytic site of the C-terminal domain (residues 209–561). Most of the pathogenic mutations reported in the ASNS protein are located in the C-terminal domain (Table 1).

The underlying pathomechanisms of ASNSD that cause its characteristic phenotype are not well understood. Asparagine is traditionally defined as a nonessential amino acid because even in the absence of dietary intake, sufficient amounts can be generated from the substrates glutamine and aspartate via ASNS. Children with ASNSD are born with microcephaly or simplified gyral pattern, indicating that significant brain damage occurred during embryonic development. ASNS was shown to be essential during brain development in a gene-trap mouse model hypomorph with ~20% of the normal basal expression level of the ASNS mRNA^1^. Mutant mice showed decreased cortical thickness and enlarged lateral ventricles, similar to findings observed in patients with ASNSD. Consequently, brain ASNS activity seems relevant for the development of this organ, and deficiency in utero may limit neuronal proliferation.

Patients with ASNSD present with features of hyperexcitability, which are apparent from their propensity for seizures, hyperreflexia, and hyperekplexia. As ASNS metabolically connects the four amino acids aspartate, asparagine, glutamate, and glutamine, the lack of asparagine and the dysregulation of the balance of excitatory neurotransmitters might contribute to neuronal damage and the enhanced excitability observed in affected patients^4,13,14^.

Symptomatic treatment of seizures with commonly used antiepileptic drugs appears problematic in most reported patients with ASNSD. Therefore, a trial performed with selective drugs that influence specific receptors of excitatory neurotransmitters could be a promising approach. Amantadine, memantine, and riluzole all target the glutamatergic system, especially NMDA receptors, and are already in use or being tested in clinical studies for other neurodegenerative diseases^26,27^. Other subgroups of glutamate receptors could also serve as a target. For example, perampanel is an antiepileptic drug that targets the AMPA receptor as a selective noncompetitive antagonist and has been approved as an adjunct treatment in patients with epilepsy older than 12 years old^28^. These drugs could offer a novel treatment modality in view of the limited efficacy of the existing anticonvulsive therapy in children with a deficiency in ASNS.

ASNSD is the third of three known disorders that affect the synthesis of nonessential amino acids (the others are serine biosynthesis disorder and glutamine synthetase deficiency)^1,29–31^. All three known deficiencies in amino acid biosynthesis mainly present with neurological features. Under disease conditions, the deficient amino acid becomes essential. Hence, a logical first consideration for therapy is dietary supplementation to provide the deficient amino acid to the brain.

Plasma levels of amino acids can usually be substantially increased by dietary supplementation^29–31^. Despite the complex transport systems that move amino acids across the blood-brain barrier, a therapeutic benefit of supplementation has already been reported in serine biosynthetic disorders and glutamine synthetase deficiency^29–31^. However, asparagine uptake across the blood-brain barrier is low, and dietary amino acid supplement therapy is therefore limited. In healthy young children, the CSF concentration of asparagine has been reported to be only 8–13% of the level found in plasma; thus, asparagine transport across the blood-brain barrier may be a limiting factor in the absence of sufficient brain ASNS enzymatic activity^13,22^. The poor transport of asparagine across the blood-brain barrier suggests that the brain depends on its local de novo synthesis, and this may explain why the phenotype is essentially neurological. Artificially elevated blood asparagine levels may even inhibit the uptake of other amino acids due to competition for shared transporters, potentially leading to secondary shortages of those within the CNS^2,32^.

The first trial of asparagine supplementation was documented in one patient with ASNSD by Alrifai et al. 2016^4^. The treated patient was a 5.5-year-old boy with severe developmental delay and no interactions with people or his surroundings, seizures intractable to multiple antiepileptic drugs, and a low asparagine level in the CSF. He harbored the homozygous missense mutation NM_133436.3 (*ASNS*_v001): c.1193A>G; p.(Tyr398Cys); and the case was reported by Alfadhel et al. 2015^6^. After administration of l-asparagine supplement at a dose of 500 mg once daily (20 mg/kg/day), the patient’s mental status improved slightly from a vegetative state to a minimally conscious state, but after a few days, the patient became irritable, developed sleep disturbance, and experienced worsening seizures. After the dose was increased to 500 mg twice a day, the seizures became even more frequent and severe, leading to discontinuation of the treatment after 27 days. The authors speculate that the flare-up of seizures might have been due to the metabolism of asparagine to aspartate, resulting in an excess of aspartate and glutamate. Another explanation for the worsening of the seizures could be the natural course of the disease, and the flare-up of seizures observed after the introduction of asparagine may have been simply coincidental.

However, the authors emphasize that no firm conclusions can be drawn from the results observed in this single case that was treated late in the disease course, and they remark that their findings cannot be generalized^4^.

In contrast, our patients tolerated supplementation with l-asparagine well without any exacerbation of seizures. EEG recordings showed that there were intermittently fewer epileptiform discharges than were observed on a baseline EEG in the sleeping state (Supplementary Fig. 1). Furthermore, we observed mild developmental progress in one of the siblings, reflected by increased attention, communication, and interaction with the environment, which was reported by the parents.

Symptomatic treatment options currently available for patients suffering from ASNSD show a very limited efficacy. Therefore supplementation with asparagine seems a reasonable therapy for ASNSD, even if the prenatal onset of microcephaly and the early postnatal presentation of symptoms related to severe epileptic encephalopathy suggest that significant and possibly irreversible tissue damage occurs during the earliest stages of development^1^. Hence, it is reasonable to assume that supplementation with asparagine will not effectively ameliorate symptoms unless started prenatally.

As such, our findings cannot be generalized. To confirm or reject our observations, further studies with more patients are needed to assess the effects of asparagine supplementation on the course of the disease.

In summary, we report a novel missense mutation in two patients with an inherited defect in the ASNS gene detected by WES and compare their phenotypic characteristics with those observed in previously reported cases. Supplementation with l-asparagine was associated with further developmental progress in our index patient, and no adverse effects occurred during treatment. Therefore, the substitution of l-asparagine should be considered in patients with an inborn error in *ASNS*. Further trials including larger cohorts are needed to investigate the effects of l-asparagine supplementation in early childhood and to determine to what extent earlier intervention might prevent the devastating natural course of ASNSD.

It is important to consider that this inborn error of metabolism is not detectable in currently available routine metabolic testing. Therefore, newborns with a matching phenotype should be considered candidates for a rapid genetic workup with next-generation sequencing.

## Declarations

SC takes full responsibility for the data, the analyses and their interpretation and the conduct of the research. He has full access to all of the data and has the right to publish any and all data separate and apart from any sponsor. He declares that all authors have approved the manuscript for submission. The authors declare that the manuscript conforms to the journal’s policies. The authors confirm that the content of the manuscript has not been published or submitted for publication elsewhere.

## Consent for publication

Written informed consent was obtained from all patients. An authorization-for-disclosure of the figures has been provided.

## Supplementary Information

Supplemental material

## Data Availability

The datasets supporting the conclusions presented in this article are included within the article and its additional file.
