# Supplementary material for: Clinical outcomes of two patients with a novel pathogenic variant in ASNS: response to asparagine supplementation and review of the literature
Source: Hum Genome Var. 2019 May 22;6:24. doi: 10.1038/s41439-019-0055-9 (PMC6531480; doi:10.1038/s41439-019-0055-9)
Supplement: Supplementary file 1 — Supplemental material [file 41439_2019_55_MOESM1_ESM.docx]

**Supplementary information:** Clinical outcome of two patients with a novel pathogenic variant in *ASNS*: response to asparagine supplementation and review of the literature.

**Supplementary table 1: Results of the variant filtering**

This chart shows the results of the variant filtering and the specific criteria we applied to the data set.

|  | Runs of homozygosity::30404 |
| --- | --- |
| Rare functional variants (RFV) | 25 |
| RFVs in good sequence quality (Q>100) | 24 |
| Non-synonymous coding, indels (with intronic mutations close to splice site < 20 base pairs up/downstream) | 24 |
| Non-reported RFVs *(*reported RFV: present in in-house, reported SNPs)* | 17 |
| Non-polymorphism predictions | 11 |
| RFVs related to central nervous movement disorders, RFVs have high expression on CNS | 5 |
| RFVs show autosomal-recessive inheritance | 3 |

WES was performed to uncover the pathogenic variant in the index patient 1.

Data analysis was performed with the Varbank V.2.25 exome pipeline of the Cologne Centre for Genomics (https://varbank.ccg.uni-koeln.de/). Sequencing data were filtered for rare (minor allele frequency <0.1%) and homozygous variants (allele read frequency of 75–100%) in accordance with the expected autosomal recessive mode of inheritance with the consanguineous familiar background.

Variants affecting protein sequence or splice sites were considered. We used the best-practice filtering scheme based on the American College of Medical Genetics and Genomics (ACMG) guidelines^1^ and common online prediction tools for variant interpretation (MutationTaster (http://www.mutationtaster.org/), PROVEAN (http://provean.jcvi.org/), and MutPred2 (http://mutpred2.mutdb.org/)). Sanger sequencing was performed for confirmation of cosegregation of the variants.

From a total number of 25 homozygous and rare functional variants, only three genetic variants fitted the filter criteria and were located to runs of homozygosity corresponding to 304 Mb in the patient (see supplementary table 1). These three variants were missense mutations in *ASNS* (NM_133436.3, c.1108C>T, p.L370F), *plexin B2* (*PLXNB2,* NM_012401.3, c.1817C>T, p.T606M) and the kinesin family member 20B (*KIF20B*, NM_016195.2, c.1865_1866delAGinsCA, p.E622A).

*PLXNB2* is a transmembrane receptor that participates in axon guidance and cell migration and for the variant, there are eleven heterozygous carriers in the Genome Aggregation Database (http://gnomad.broadinstitute.org/) reported. PLXNB2 expression is upregulated in gliomas,^2^ but no further disease has been associated with it. We classified this mutation as “uncertain significance” by the standards and guidelines of the ACMG.

*KIF20B* encodes a plus-end-directed motor enzyme required for completion of cytokinesis and 16 homozygous carriers of the mutation p.E622A are reported in the Genome Aggregation Database. We classified this variant as “likely benign” by the ACMG standards.

The deficiency in ASNS is described in Orphanet (https://www.orpha.net/) as a syndrome with congenital microcephaly, severe encephalopathy, and progressive cerebral atrophy. This depiction fits overtly to our phenotype.

**Multiple sequence alignment**

ASNS multiple sequence alignment was performed between selected orthologues by using the NCBI Standard Protein BLAST platform. The alignment was visualized and analyzed with Jalview 2.10.5 online tool.^3^

**Protein modeling**

The 3D ASNS protein structure was homology-modeled using the SWISS-MODEL online server (https://swissmodel.expasy.org/).^4^ ASNS wildtype and mutant p.Leu370Phe FASTA sequences were modeled based on the crystal structure of asparagine synthetase B from Escherichia coli (PDB 1CT9.1.A) as a template.^5^ The top-ranking models were downloaded and visualized using RasMol Molecular Graphics Visualization Tool (http://www.openrasmol.org/).

The linear model of the human ASNS protein was visualized with the Domain Graph software version 1.0.^6^


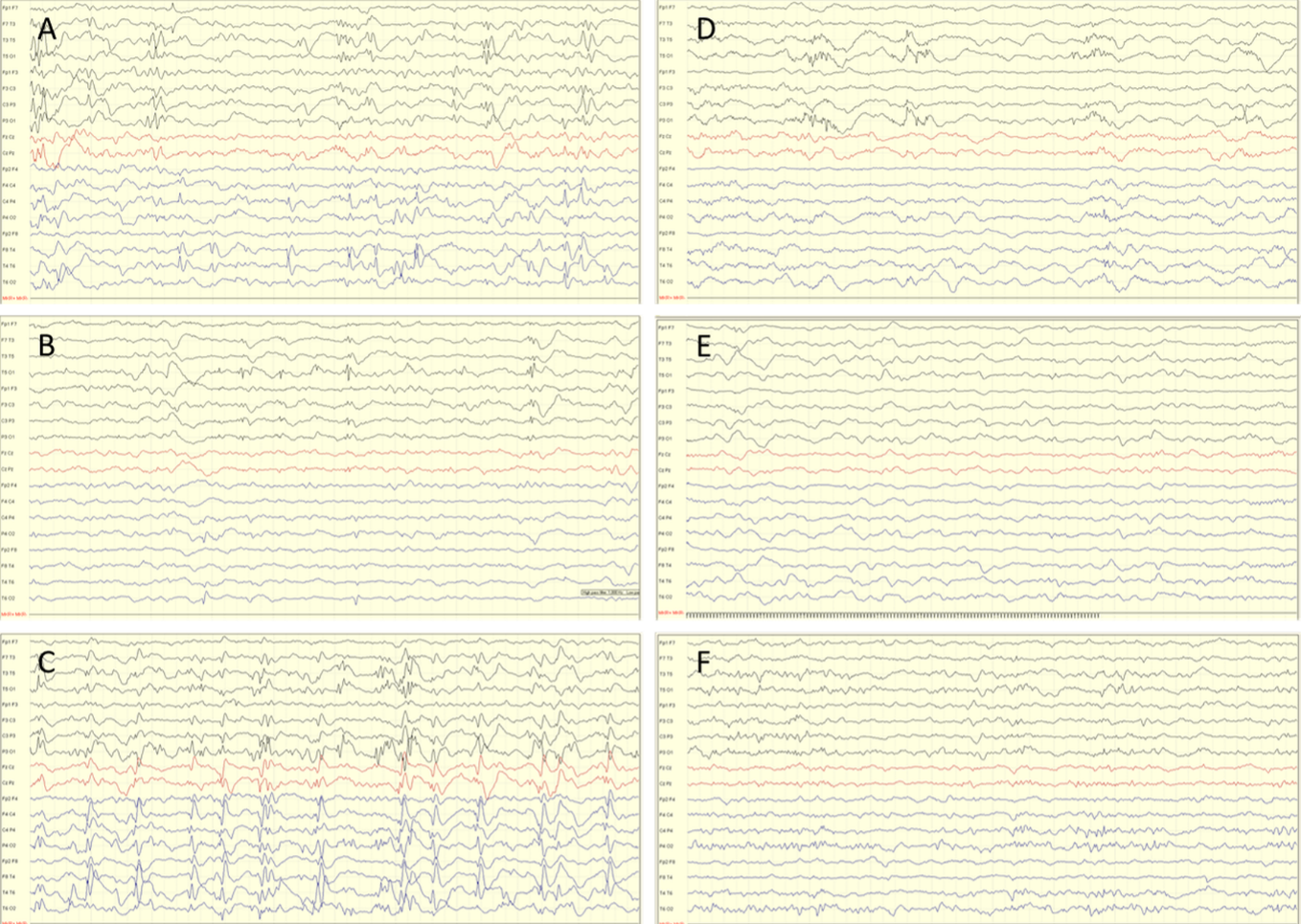


**Supplementary Figure 1: EEGs before and after 6 and 21 months of treatment.** Filter settings: Low frequency 1 Hz, high frequency 70 Hz, amplitude 200 µV/cm, 10 sec/page.

(A) Sleep EEG of patient 1 at 3 years, prior to asparagine supplementation, shows intermittent slow background activity with multifocal epileptiform discharges with spikes and sharp waves. (B) Sleep EEG of the patient 1 at the age of 4 years and 3 months after 6 months of asparagine supplementation shows low amplitude and slow background rhythm with intermittent left temporal and parietooccipital spikes and sharp waves. Note the reduction of epileptiform discharges compared to the baseline EEG. (C) Sleep EEG of patient 1 at 5.5 years after 21 months of asparagine treatment shows numerous multifocal irregular spikes and sharp waves with a maximum in posterior regions and an increased frequency compared to the previous EEG.

(D) Sleep EEG of patient 2 at the age of 1.5 years, prior to asparagine supplementation, shows intermittent slow background rhythm with rare bilateral posterior small spikes and sharp waves, accentuated on the left side. (E) Sleep EEG of patient 2 at 2 years and 3 months after 6 months of asparagine supplementation shows low amplitude and slow background rhythm without epileptiform discharges. (F) EEG of patient 2 at 3.5 years of age after 21 months of treatment with asparagine shows low amplitude and superimposed fast activity with infrequent isolated sharp waves over both posterior hemispheres.

**References**

1. Richards S, Aziz N, Bale S, Bick D, Das S, Gastier-Foster J *et al.* Standards and guidelines for the interpretation of sequence variants: a joint consensus recommendation of the American College of Medical Genetics and Genomics and the Association for Molecular Pathology. 2015;17:405-424.

2. Le AP, Huang Y, Pingle SC, Kesari S, Wang H, Yong RL *et al.* Plexin-B2 promotes invasive growth of malignant glioma. Oncotarget 2015;6:7293-7304.

3. Waterhouse AM, Procter JB, Martin DM, Clamp M, Barton GJ. Jalview Version 2 - a multiple sequence alignment editor and analysis workbench. Bioinformatics (Oxford, England) 2009;25:1189-1191.

4. Waterhouse A, Bertoni M, Bienert S, Studer G, Tauriello G, Gumienny R *et al.* SWISS-MODEL: homology modelling of protein structures and complexes. Nucleic acids research 2018;46:W296-w303.

5. Larsen TM, Boehlein SK, Schuster SM, Richards NG, Thoden JB, Holden HM *et al*. Three-dimensional structure of Escherichia coli asparagine synthetase B: a short journey from substrate to product. Biochemistry 1999;38:16146-16157.

6. Ren J, Wen L, Gao X, Jin C, Xue Y, Yao X. DOG 1.0: illustrator of protein domain structures. Cell research 2009;19:271-273.
